# Parametric mapping using spectral analysis for ^11^C-PBR28 PET reveals neuroinflammation in mild cognitive impairment subjects

**DOI:** 10.1007/s00259-018-3984-5

**Published:** 2018-03-09

**Authors:** Zhen Fan, Melanie Dani, Grazia D. Femminella, Melanie Wood, Valeria Calsolaro, Mattia Veronese, Federico Turkheimer, Steve Gentleman, David J. Brooks, Rainer Hinz, Paul Edison

**Affiliations:** 1 Department of Medicine, Neurology Imaging Unit, Imperial College London, London, W12 0NN UK; 2 Department of Neuroimaging, Institute of Psychiatry, Kings College London, London, WC2R 2LS UK; 3 Department of Nuclear Medicine, Aarhus University, Aarhus C, Denmark; 4 Wolfson Molecular Imaging Centre, University of Manchester, Manchester, UK

**Keywords:** Spectral analysis, ^11^C–PBR28, PET, MCI, Compartmental modelling, Logan graphic analysis

## Abstract

**Purpose:**

Neuroinflammation and microglial activation play an important role in amnestic mild cognitive impairment (MCI) and Alzheimer’s disease. In this study, we investigated the spatial distribution of neuroinflammation in MCI subjects, using spectral analysis (SA) to generate parametric maps and quantify ^11^C–PBR28 PET, and compared these with compartmental and other kinetic models of quantification.

**Methods:**

Thirteen MCI and nine healthy controls were enrolled in this study. Subjects underwent ^11^C–PBR28 PET scans with arterial cannulation. Spectral analysis with an arterial plasma input function was used to generate ^11^C–PBR28 parametric maps. These maps were then compared with regional ^11^C–PBR28 V_T_ (volume of distribution) using a two-tissue compartment model and Logan graphic analysis. Amyloid load was also assessed with ^18^F–Flutemetamol PET.

**Results:**

With SA, three component peaks were identified in addition to blood volume. The ^11^C–PBR28 impulse response function (IRF) at 90 min produced the lowest coefficient of variation. Single-subject analysis using this IRF demonstrated microglial activation in five out of seven amyloid-positive MCI subjects. IRF parametric maps of ^11^C–PBR28 uptake revealed a group-wise significant increase in neuroinflammation in amyloid-positive MCI subjects versus HC in multiple cortical association areas, and particularly in the temporal lobe. Interestingly, compartmental analysis detected group-wise increase in ^11^C–PBR28 binding in the thalamus of amyloid-positive MCI subjects, while Logan parametric maps did not perform well.

**Conclusions:**

This study demonstrates for the first time that spectral analysis can be used to generate parametric maps of ^11^C–PBR28 uptake, and is able to detect microglial activation in amyloid-positive MCI subjects. IRF parametric maps of ^11^C–PBR28 uptake allow voxel-wise single-subject analysis and could be used to evaluate microglial activation in individual subjects.

**Electronic supplementary material:**

The online version of this article (10.1007/s00259-018-3984-5) contains supplementary material, which is available to authorized users.

## Introduction

Amnestic mild cognitive impairment (MCI) is a transitional stage between preclinical Alzheimer’s disease and dementia. Microglial activation plays a significant role in Alzheimer’s disease, along with amyloid and tau deposition [1–3]. Recent PET imaging studies have suggested that microglial activation correlates closely with the severity of dementia [1, 4, 5]. However, imaging microglia has been challenging. The 18-kDa translocator protein (TSPO) is a cholesterol-transporter protein expressed in the outer mitochondrial membrane of microglial cells and astrocytes in the brain. TSPO expression in normal brain is very low, but it increases significantly after trauma and inflammation [6–8]. The PET tracer ^11^C–R-PK11195 PET has been used for over 20 years to assess the level of TSPO expression and microglial activation [9–11]. However, ^11^C–R-PK11195 has a poor signal-to-noise ratio [6, 12]. In an attempt to improve the signal-to-noise ratio, several second-generation TSPO radioligands have been developed. The new-generation TSPO tracers are affected by genetic variability of TSPO binding site induced by the rs6971 single-nucleotide polymorphism [13], and recent studies have demonstrated that tracer signal in the high-affinity binders (HAB) is 25–35% higher than in the mixed affinity binders (MAB) [14, 15]. However, our group has previously demonstrated that results gathered from a TSPO subgroup (HAB or MAB) can be translated to the entire AD and MCI population [16].

^11^C–PBR28 is a second-generation TSPO PET tracer, and can be used to quantify microglial activation in neurodegenerative disease; however, recent studies have demonstrated discordant results in neuroinflammation in AD or MCI subjects using ^11^C–PBR28 PET imaging [5, 17–19]. In fact, the quantification of TSPO is challenging; the tracer binds not only to microglia (and to a lesser extent to astrocytes) in the parenchyma but also to the endothelium and smooth muscle cells [20–22]. Since endothelial TSPO is physically in contact with plasma, its apparent affinity for radioligands is higher than parenchymal TSPO.

Spectral analysis (SA) is a powerful kinetic tool for generating parametric maps of ligand volumes of distribution from brain TACs with a plasma input function. As a spectral technique, it makes no assumptions about kinetic compartments, but identifies heterogeneous kinetic components which represent tracer delivery, vascular binding, and later parenchymal tracer-binding components [22, 23].

In this study, for the first time, we performed spectral analysis on ^11^C–PBR28 PET dynamic images to generate parametric maps of ligand uptake reflected by area under impulse response function (IRF). We compared the SA parametric mapping to the compartmental models and the parametric mapping using Logan graphic analysis. Finally, we evaluated microglial activation between two cohorts (MCI vs HC) using ^11^C–PBR28 SA parametric mapping, Logan V_T_ and two tissue compartment models, with a view to identify the most appropriate quantification approach for ^11^C–PBR28 PET in neurodegenerative diseases.

## Materials and methods

### Demographics

Thirteen MCI patients and nine age-matched healthy controls (all genetically high binders for TSPO ligands — HAB) were recruited from memory clinics and the Join Dementia Research website. This study was approved by the local and regional regulatory ethics committee (London Riverside Research Ethics Committee - National Health Research Services, Health Research Authority, UK), and the approval for administration of radioactivity was given by ARSAC (Administration of Radioactive Substances Advisory Committee).

### Image acquisition

MRI scans were acquired for all subjects with a 3 Tesla SIEMENS 32-channel Verio MRI scanner (MPRAGE; time repetition = 2400 ms, time echo = 3.06 ms, flip angle of 9, inversion time = 900 ms, matrix = 256 × 246). The T1 images were used for co-registration of the PET for ROI analysis, while T2-weighted images were used to exclude any significant white matter microvascular disease.

All subjects underwent ^11^C–PBR28 PET scans with a SIEMENS Biograph TruePoint PET/CT scanner (axial field = 21.8 cm, transaxial planes =111, and spatial resolution = 2.056 mm × 2.056 mm × 2 mm) at Imanova, London. A low-dose CT scan was performed for attenuation correction, followed by injection of a mean activity of 300 MBq ^11^C–PBR28 intravenously. A continuous 3D dynamic acquisition was performed in list mode for 90 min. The dynamic ^11^C–PBR28 PET data were reconstructed using scatter correction, attenuation correction and random correction. Then the data were corrected for decay and rebinned as 26 time frames.

Twenty-one subjects (12 MCI and 9 HC) also had ^18^F–Flutemetamol PET scans using a Siemens Biograph 6 PET/CT scanner. A mean activity of 182 (±2.5) MBq ^18^F–Flutemetamol was injected intravenously. PET data were acquired 90–120 min after the injection to generate a static 3D ^18^F–Flutemetamol PET image. The cerebellum was used as the reference region to create the ^18^F–Flutemetamol RATIO image as previously described [24].

### Blood data

All patients had continuous online blood sampling for the first 15 min after the ^11^C–PBR28 PET scan started, and 12 discrete blood samples were taken at 5, 10, 15, 20, 25, 30, 40, 50, 60, 70, 80, and 90 min, allowing blood and plasma radiotracer activity to be measured. The time course of ^11^C–PBR28 activity in the plasma was calculated with a linear model fit using plasma-to-blood ratio model in the first 15 min of online whole-blood data. Parent tracer and metabolite levels were measured using HPLC analysis for discrete blood samples. A sigmoid model was applied to describe the parent fraction of ^11^C–PBR28, which was then used to generate the parent fraction of plasma input function.

### Quantification of ^11^C–PBR28 PET using spectral analysis

Spectral analysis applies a positively constrained general linear model to fit the tissue kinetics with a large matrix of exponential functions with a range of decaying factors convoluted with the plasma input function [25]. The solution to each fit is a vector of linear coefficients αi (peak height), each corresponding to a decay βi (peak position), and is obtained using non-negative least squares (NNLS) [23, 26]. In the solution vector, the non-zero coefficients for the high-frequency components (e.g., large βi) usually reflect the dynamics of the tracer in the blood; the ones with slower βi reflect the kinetics of reversible parenchymal perfusion between plasma or tissue compartments, while the slowest components represent irreversible trapping. The beta-min was used as the decay constant of the ^11^C (0.00056629) for ^11^C–PBR28. The tissue ^11^C–PBR28 ligand-binding response could be measured as portion of impulse response function (IRF), which was reflected by the sum of the intermediate and low frequency components of the spectrum. In this study, we generated IRF(t) parametric maps with different epochs of observation-time: 30 min, 45 min, 60 min, 75 min, and 90 min for ^11^C–PBR28 using the formula:

$$ IRF(t)=\sum \limits_{i=1}^{\mathrm{n}}{\alpha}_i\ast {e}^{-\left({\beta}_i-\lambda \right)\ast t} $$

where t represents the selected observation time, n denotes number of PET time frames, αi and βi are the peak height and peak position for time frame i. We used a decay constant λ of 0.00056629 for ^11^C–PBR28. In order to provide a high quality IRF(t) parametric maps, the coefficient of variation (CV) (standard deviation/mean) of ^11^C–PBR28 binding in major cortices (frontal, temporal, parietal, and occipital lobe) and thalamus were calculated at different observation time; the lower CV indicated a higher precision and lower noise level for that IRF(t) parametric map. (Online Resource 1A).

### Quantification of ^11^C–PBR28 PET using compartmental modelling

Compartmental modelling of TACs was performed to determine the volumes of distribution of the ^11^C–PBR28 tracer using the parent plasma input function and dynamic PET acquisition. In this study, we fitted kinetic data to two compartmental models for ^11^C–PBR28 [21, 27]: the two-tissue compartmental model (2TCM4k), and the two-tissue compartmental model with an extra vascular component (2TCM4k-1 K), which accounts for the extra binding compartment for the endothelial cells of blood vessels [21, 28]. We calculated the Akaike information criterion (AIC) to evaluate the model performance [29, 30]. The model with lower AIC was chosen as the preferred model for a predefined ROI. Total volume of distribution (V_T_), rate constant (k), and binding potential (BP_ND_) were the parameters derived from the kinetic models using MICK software (modelling input function compartmental kinetics) and MATLAB2014a.

### Quantification of ^11^C–PBR28 PET using Logan graphic analysis

Logan analysis is a graphical quantification method which linearises PET uptake data using a plasma or non-specific tissue reference input function where ligands bind reversibly. The gradients of fits represent V_T_ and can be used for parametric mapping at a voxel level, though measurement noise is also linearised and a potential confound. Logan graphic analysis is independent of the number of compartments as long as they are all in equilibrium after a time t. The ^11^C–PBR28 Logan V_T_ parametric map was generated, where V_T_ reflects the slope of the linear section of the Logan graphic plot [31]. Logan graphical analysis was performed using MICK parametric mapping software developed in MATLAB2014a. CV was measured in major cortices and thalamus to assess the signal variation for the Logan V_T_.

### Region of interest (ROI) analysis in MCI and HC

Regional quantification of mean ^11^C–PBR28 binding was estimated for compartmental model V_T_, SA parametric map (IRF) and Logan V_T_ map in MCI and healthy controls. To evaluate the binding, MRI and brain atlas (83-region Hammersmith atlas) were spatially transformed into native PET space using SPM8, and Analyze 11.0 (AnalyzeDirect) was used to sample the regional mean tracer update in predefined ROI regions as described in our previous studies. The ROI regional mean uptake was sampled in the following regions: posterior cingulate, frontal, temporal, parietal, and occipital lobes. ROI analysis was also performed for medial temporal lobe, hippocampus, and thalamus. The regional ROI value was regarded as significantly raised (positive) for that region when it is elevated more than 2SD above the mean of healthy controls. The MCI subjects were considered as ^11^C–PBR28 positive when at least one cortical region was positive.

### Voxel-level analysis between MCI and HC

The parametric maps generated by SA or Logan graphic analysis allowed us to perform voxel-wise comparisons between patients and healthy controls. 1) Parametric maps were co-registered to their corresponding MRIs, 2) normalization and smoothing (6 mm × 6 mm × 6 mm) were applied to the co-registered PET to generate the individual normalized PET in SPM8, and 3) each patient’s normalized parametric map was then compared against a group of controls to generate a single-subject SPM T-map at voxel level, which localised significant pathological increases in each patient. The significant cluster threshold was set at *p* < 0.05 with an extent threshold of 50 voxels. Family-wise error rate (FWE) was corrected for multiple comparisons. For each subject who had significant clusters of increased ^11^C–PBR28, those clusters were extracted as individual VOI (volume of interest) maps, and the total volume of VOI was calculated in mm^3^ in Analyze 11.0.

### Brain amyloid status

As the MCI cohorts are heterogeneous, the brain amyloid status for each subject was evaluated from ^18^F–flutemetamol RATIO images. In this study, an amyloid-positive subject was defined as having uptake in at least one of the large cortices or two separate cortical regions higher than the mean + 2SD of healthy controls.

### Statistics

Group mean and standard deviation were calculated in SPSS23 (SPSS, Chicago, IL, USA), for each diagnostic group, and Student’s *t*-test was used to interrogate significant increases in the tracer uptake. The Pearson correlation coefficient was applied to measure the degree of linear correlation between two groups of variables using SPSS23, and a *p*-value of < 0.05 was regarded as a significant correlation.

## Results

### Demographics

The demographic details of 13 MCI and 9 control subjects (aged 54–79 yrs) are detailed in the Online Resource 1B. The MCI cohort demonstrated significantly lower neuropsychometric test scores compared to the age-matched healthy controls.

### Brain amyloid status

^18^F–Flutemetamol scans were performed for 12 MCI and 9 HC subjects. Seven of the 12 MCI subjects had significantly higher amyloid deposition than healthy controls in multiple brain regions, and were classified as amyloid-positive.

### Spectral analysis

**Fig. 1:**
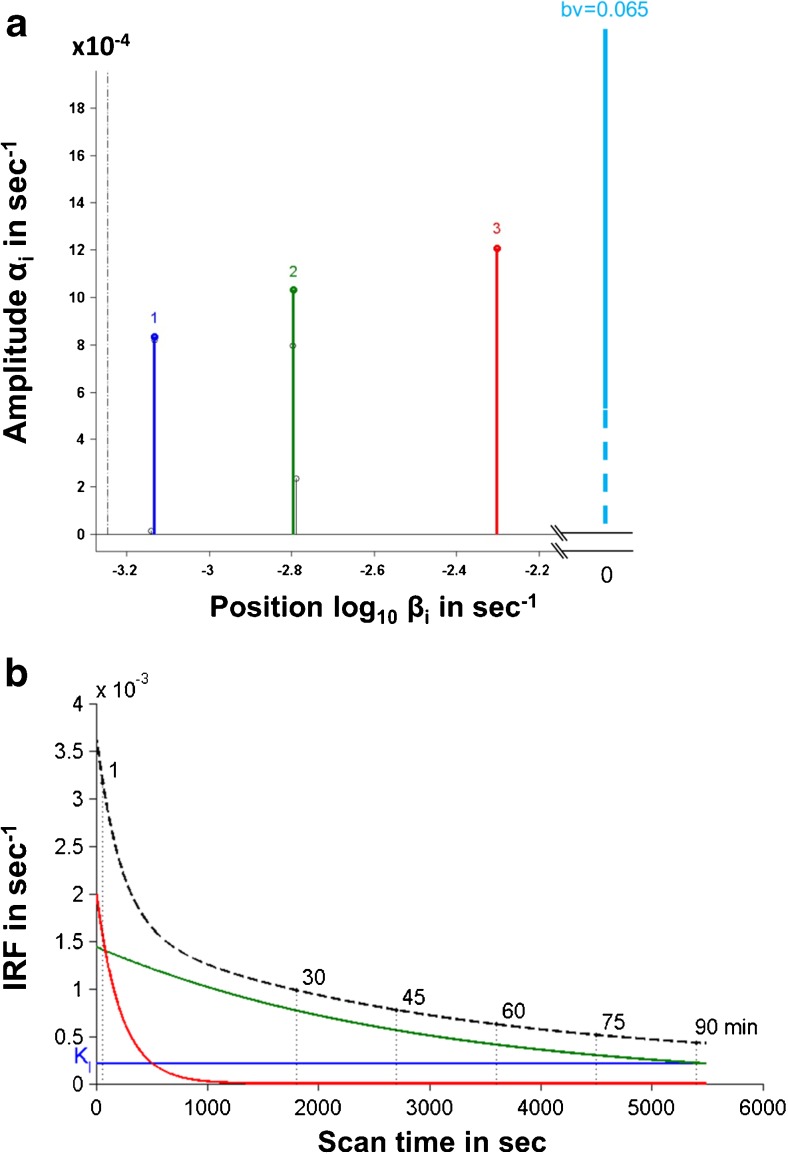
**a** Kinetic spectrum for a healthy control subject, which revealed three different components [at position βi of 7.07e-04 s^-1^ (*blue*), 1.58e-03 s^-1^ (*green*) and 5.01e-03 s^-1^ (*red*) with amplitude αi of 8.35e-04 s^-1^, 1.03e-03 s^-1^, and 1.21e-03 s^-1^] with fractional blood volume (*bv*) of 0.065 (*cyan*). **b** Predicted curves using spectral analysis IRF (impulse response function) for tracer activity (*dashed line*) which was measured by the sum of three individual components of the spectrum. The *blue* (K_I_), *green* and *red* curves corresponded to the three component peaks in the spectrum

We identified three peaks along with fractional blood volume of 0.065 (± 0.013) in spectra (Fig. 1a). The high-frequency kinetic spectral component probably corresponded to the vascular TSPO (see Turkheimer et al., 2007 [20]) while the other two probably represented free and bound tracer in parenchyma. The tissue IRF(t) curve is equivalent to the sum of three spectral component peaks (Fig. 1b). Given different sets of observation time for generating IRF parametric map, IRF at 90 min (IRF-90) demonstrated the lowest noise level with global CV mean of 18% (SD = 4%).

**Fig. 2:**
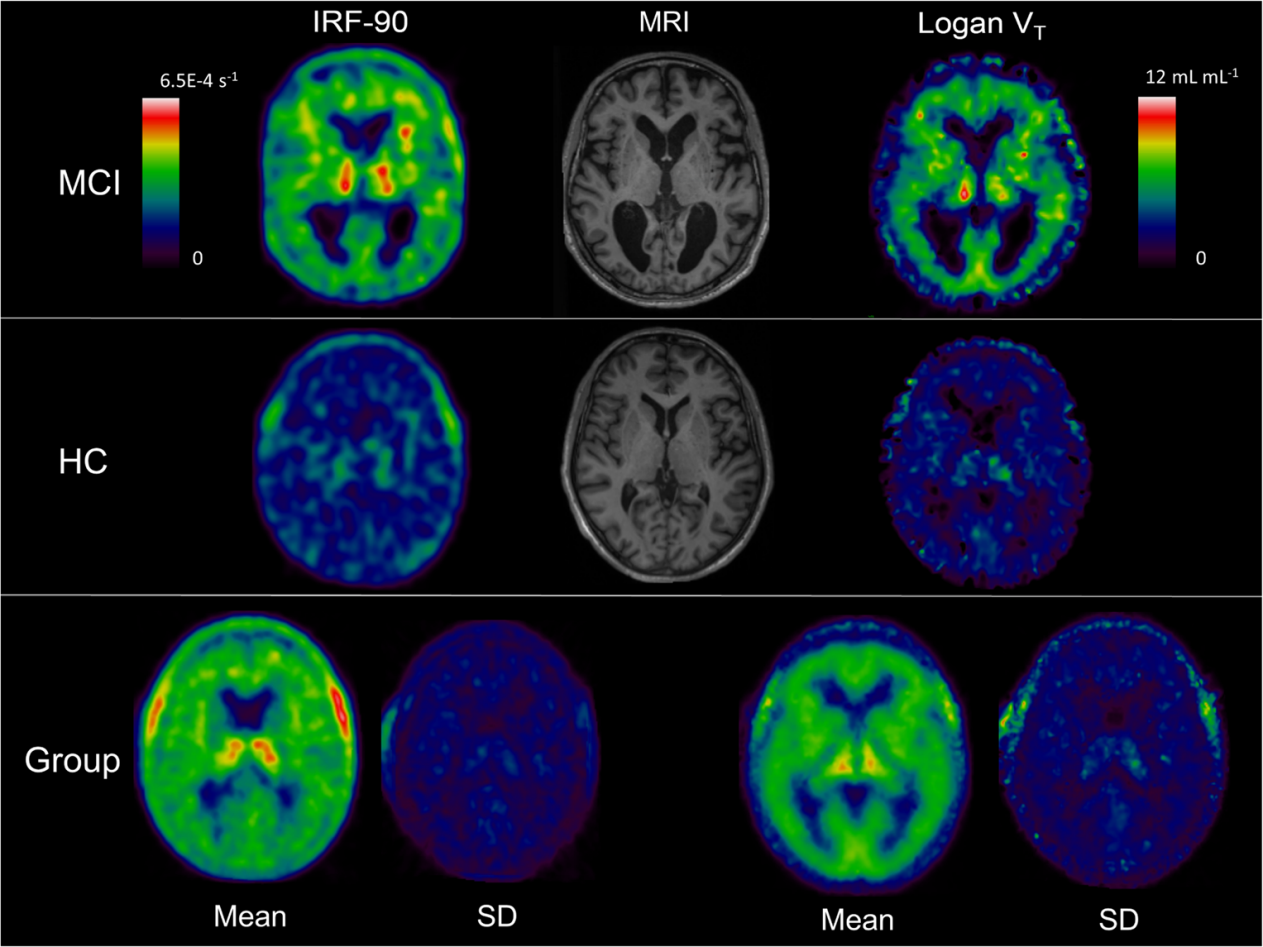
Individual IRF-90 and Logan V_T_ parametric maps demonstrated with corresponding MR image. *Upper panel* displays an *MCI* patient, *middle panel* shows a healthy control (*HC*), and *lower panel* demonstrates the group-wise average image (*Mean*) and standard deviation image (*SD*). The *colour bar on the left* represents the colour scale used for IRF-90 images, and the *colour bar on the right* represents the colour scale used for Logan V_T_ images

**Fig. 3:**
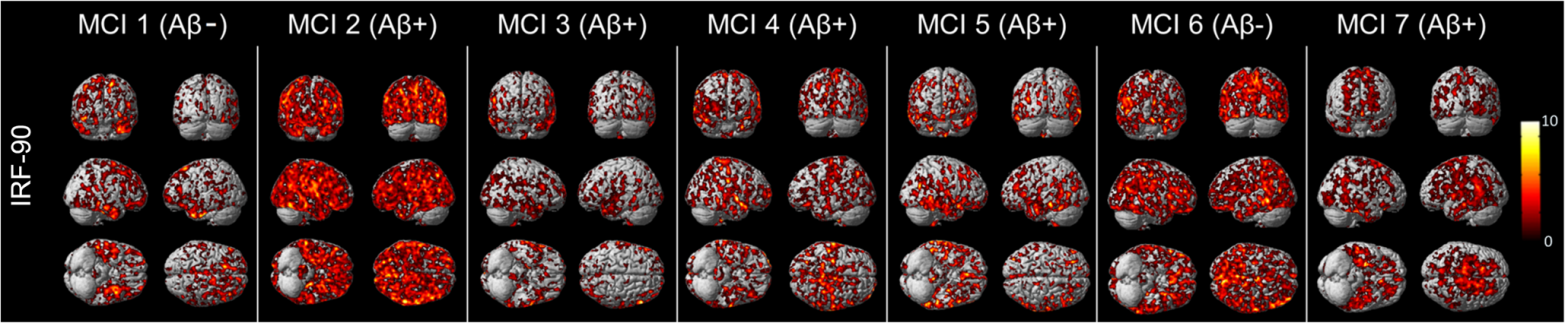
Single-subject VOI analysis of IRF. The *colour map* represents the significant clusters of increased ^11^C–PBR28 binding for each MCI patient compared to healthy control cohort. *Aβ +* and *Aβ-* represent amyloid-positive MCI and amyloid-negative MCI subjects respectively. The *colour bar* indicates the significant Z-score which was used for the colour-coded map

The ^11^C–PBR28 IRF parametric map (Fig. 2) generated by spectral analysis provided the opportunity to evaluate each patient for neuroinflammation at a voxel level. In this study, the single-subject analysis detected seven MCI patients (five amyloid-positive and two amyloid-negative) who had significant clusters of elevated microglial activation in frontal gyrus, temporal lobe, parietal gyrus, anterior cingulate gyrus, occipital lobe and in thalamus compared to the healthy control cohort (Fig. 3 and Online Resource 2). Furthermore, VOI analyses were performed on those seven MCIs who had significantly increased ^11^C–PBR28, which measured volumes of significantly increased ^11^C–PBR28 binding ranging from 114,982 to 714,801mm^3^ (Table 1).

**Table 1:** The volume of significant ^11^C–PBR28 increase in single subject using ^11^C–PBR28 parametric mapping of IRF and Logan graphic analysis

Subject	Increased ^11^C–PBR28 volume (mm^3^)	
	IRF	Logan
MCI 1	191,073	\
MCI 2	714,801	423,979
MCI 3	114,982	\
MCI 4	260,442	165,193
MCI 5	177,119	\
MCI 6	360,328	289,142
MCI 7	373,282	108,237

In the ROI analysis, parametric mapping of ^11^C–PBR28 IRF revealed a group-wise significant increase in neuroinflammation in the temporal lobe and multiple other brain regions in the amyloid-positive MCI subjects compared to healthy controls (Table 2). Individually, four amyloid-positive MCIs and two amyloid-negative MCIs revealed ROIs with higher binding than mean + 2SD of healthy controls.

**Table 2:** ^11^C–PBR28 ROI group results of IRF-90 mean and standard deviation in amyloid positive MCI subjects and healthy controls

	Healthy control		Amyloid-positive MCIs			
	Mean	SD	Mean	SD	*P* value	%
Frontal lobe	0.00037	0.00008	0.00045	0.00008	*0.074*	*19%*
Temporal lobe	0.00039	0.00006	0.00048	0.00008	0.010*	*25%*
Parietal lobe	0.00035	0.00006	0.00041	0.00010	*0.115*	*16%*
Occipital lobe	0.00036	0.00005	0.00041	0.00011	*0.136*	*14%*
Post-cingulate	0.00037	0.00008	0.00048	0.00011	0.025*	*31%*
Thalamus	0.00045	0.00007	0.00057	0.00014	0.024*	*29%*
Striatum	0.00037	0.00008	0.00040	0.00008	*0.220*	*10%*
Brainstem	0.00053	0.00005	0.00062	0.00012	*0.060*	*16%*
MTL	0.00042	0.00005	0.00053	0.00008	0.005*	*27%*
Hippocampus	0.00044	0.00005	0.00054	0.00010	0.019*	*22%*
Amygdala	0.00044	0.00006	0.00057	0.00008	0.003*	*29%*
Fusiform	0.00040	0.00006	0.00053	0.00010	*0.009*	*32%*
Cerebellum	0.00037	0.00007	0.00047	0.00010	0.023*	*27%*
Whole brain	0.00037	0.00006	0.00045	0.00009	0.049*	*20%*

*P < 0.05; MTL = medial temporal lobe; SD = Standard deviation

### Compartmental modelling

**Fig. 4:**
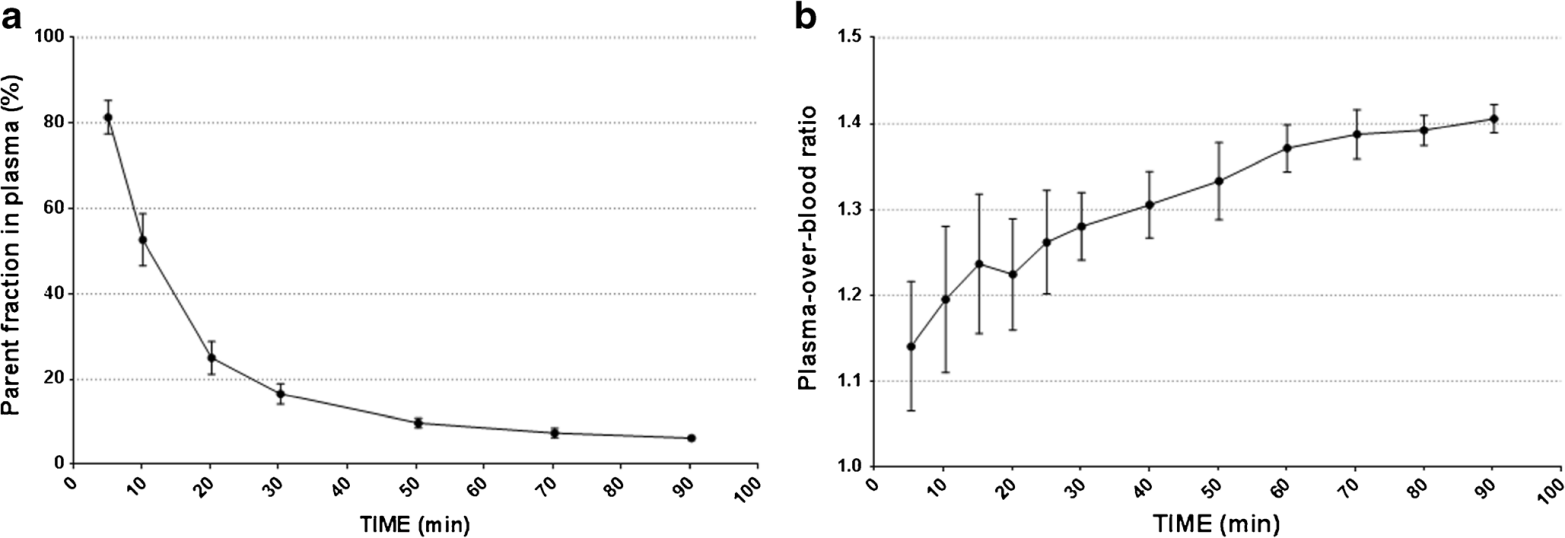
^11^C–PBR28 blood data. **a** Parent fraction in arterial plasma. **b** Plasma over blood ratio

**Fig. 5:**
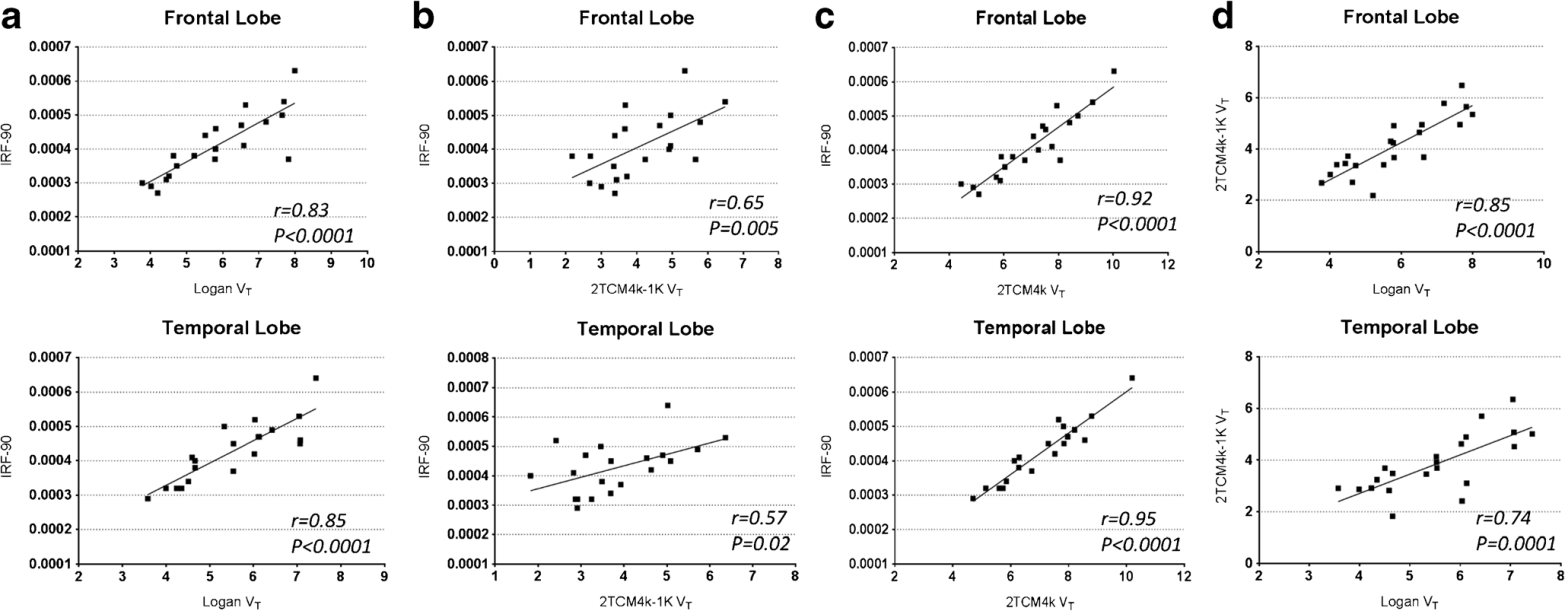
**a** The correlation between ^11^C–PBR28 IRF-90 and Logan V_T_ in frontal lobe and temporal lobe. **b** The correlation between ^11^C–PBR28 IRF and 2TCM4k-1 K V_T_ in frontal lobe and temporal lobe. **c** The correlation between ^11^C–PBR28 IRF and 2TCM4k V_T_ in frontal lobe and temporal lobe. **d** The correlation between 2TCM4k-1 K V_T_ and 2TCM4k V_T_ in frontal lobe and temporal lobe

The parent plasma fraction and the plasma:blood ratio of ^11^C–PBR28 over 90 min are shown in Fig. 4. The 2TCM4k-1 K (AIC = −84) compared with the 2TCM4k model (AIC = −76) provided a marginally better fit of ^11^C–PBR28 PET uptake data (Online Resource 3). This is consistent with previous studies [21, 32]. V_T_ derived from 2TCM4k-1 K and 2TCM4k correlated well with a Pearson correlation coefficient around 0.7 (*p* < 0.0001) (Fig. 5).

Compartmental V_T_ demonstrated a group-wise increase in thalamus (31%, *p* < 0.037) and left MTL (32%, *p* < 0.05) in ^11^C–PBR28 V_T_ in amyloid-positive MCI subjects compared to the controls. Individually, 4/7 of amyloid-positive MCI subjects showed increased uptake in ^11^C–PBR28 V_T_ compared to the controls, while one amyloid-negative MCI subject demonstrated an increase in ^11^C–PBR28 V_T_. Both compartmental models, 2TCM4k-1 K V_T_ and 2TCM4k V_T_ were positively correlated with the ^11^C–PBR28 binding in IRF parametric maps (Fig. 5). A Pearson correlation was performed to assess the relationship between 11C–PBR28 IRF-90 and BP_ND_ (k3/k4), and a positive correlation was found in frontal lobe (*r * = 0.55, *p* = 0.01), temporal lobe (*r * = 0.7, *p* < 0.0001), parietal lobe (*r * = 0.76, *p * < 0.0001), and occipital lobe (* r* = 0.57, *p* = 0.007). (Online Resource 4).

#### Logan graphic analysis

Based on linear fit of the whole brain ^11^C–PBR28 uptake and parent plasma input function, dynamic data from 2000 to 5400 s (six data points) were selected to generate Logan plot (slope = 3.8 ± 0.09 and intercept = −3033 ± 214) to create Logan V_T_ parametric map. Logan V_T_ resulted in a higher CV compared to IRF-90 parametric maps in different cortices (Fig. 2 and Online Resource 1A). With the VOI analysis, Logan maps localised a volume range of 108,237 to 423,979 mm^3^ containing increased neuroinflammation in individuals (Table 1). At a voxel level, single-subject analysis of ^11^C–PBR28 Logan parametric V_T_ maps revealed three amyloid-positive MCI subjects and one amyloid-negative MCI subject who had neuroinflammation in anterior cingulate, frontal gyrus, temporal gyrus, parietal lobe, and occipital lobe compared with healthy controls.

A good correlation was found between IRF-90 and Logan parametric maps of ^11^C–PBR28 (Fig. 5). However, Logan parametric maps failed to localise clusters of significantly raised mean ^11^C–PBR28 uptake at the group level. Individually, ROI analysis revealed two amyloid-positive and one amyloid-negative MCI subjects with a higher level of ^11^C–PBR28 binding compared with mean + 2SD of healthy controls.

## Discussion

For the first time, we have demonstrated that spectral analysis can be used reliably to quantify ^11^C–PBR28 PET. ^11^C–PBR28 PET tracer has 80-fold higher affinity for TSPO compared to previous tracers developed to evaluate microglial activation; however, different methodological approaches used in different studies have produced varying results [5, 14, 15, 33, 34]. As TSPO distribution has a heterogeneous cellular distribution with endothelial, smooth muscle, and parenchymal components all exhibiting different kinetic behaviour, in this study we evaluated the feasibility of generating ^11^C–PBR28 parametric map using spectral analysis. IRF-90 parametric mapping of ^11^C–PBR28 correlated well with Logan V_T_ and V_T_ generated by compartment models. Compared to compartmental analysis, the IRF-90 parametric maps provided model free quantification and enable comparison between subjects or between regions at a voxel level. Compared with Logan V_T_ maps, ^11^C–PBR28 IRF-90 parametric maps showed a lower noise level. IRF parametric mapping of ^11^C–PBR28 was able to reveal group-wise significant microglial activation in amyloid-positive MCI subjects across multiple brain regions. On an individual basis, IRF-90 parametric maps revealed microglial activation in individuals who had significant amyloid deposition.

As vascular binding can interfere with quantification of parenchymal binding of TSPO tracers [35], spectral analysis [22, 23] has the advantage of separating the high-frequency component which represents tracer binding in the vasculature. SA identifies two tissue components, and another low frequency component which is suggestive of an additional irreversible vascular trapping component to ^11^C–PBR28 kinetics. Interestingly this is in agreement with the spectral analysis application to ^11^C–PK11195 [20]. The time-course of changes in ^11^C–PBR28 IRF is reflected by the sum of low and intermediate frequency components in the spectrum. In this study, we have demonstrated that 90 min is optimal for generating IRF parametric mapping of ^11^C–PBR28.

It has now been established that TSPO binding is quite heterogeneous within a given area in brain. It is also suggested that TSPO could be expressed in microglia, endothelial cells, and there is a debate about the expression in astrocytes. Some studies have demonstrated that there is irreversible binding to the vascular endothelium, while there is reversible binding to the brain tissue. This gives rise to heterogeneous kinetics of both reversible and irreversible components. Spectral analysis does not make any a-priori assumptions about whether a tracer is reversible or irreversible, and provides optimal parametric map at the pixel level [22, 26]. In the spectrum, each tissue compartment is displayed as a spectral component with an amplitude (α) and a frequency (β). The impulse response function generated by the convolution of these components for ^11^C–PBR28 PET indicates the response of the brain tissue in response to the injected TSPO tracer. This could be quantified using a complex mathematical model as described below. A spectral analysis allows us to quantify the tissue response throughout the whole cortex without any a-priori assumption. Additionally, it allows us to quantify the tracer uptake in an individual subject. There is extensive evidence in brain imaging and molecular biology studies, which have demonstrated that in-vivo brain amyloid deposition is closely correlated with microglial activation in AD and MCI subjects [36–38]. Okello et al. reported that MCI patients with higher amyloid deposition had significantly higher mean levels of cortical microglial activation [39]. In our study, seven MCIs have been classified as amyloid-positive subjects using ^18^F–flutemetamol PET. Compared to other modelling methods, IRF parametric mapping of ^11^C–PBR28 has revealed a more extensive profile of neuroinflammation in amyloid-positive MCIs, with a 19–27% group increase across temporal lobe and multiple cortical regions. Compartmental analysis only demonstrated group-wise increased ^11^C–PBR28 in the amyloid-positive group in the thalamus, while Logan parametric maps failed to localise any significant increase at the group level.

Considering the heterogeneity of neuroinflammation in MCIs, it is crucial for researchers to assess the neuroinflammation on an individual basis rather than as a group. In this study, ^11^C–PBR28 IRF parametric mapping demonstrated a larger total volume of significantly increased ^11^C–PBR28, especially for MCI subjects who had amyloid plaques, consistent with the neuropathological findings. Interestingly, one amyloid-positive MCI subject, who revealed a significant cluster of increased microglial activation when IRF and Logan parametric maps were interrogated at a voxel level, was negative with an ^11^C–PBR28 ROI analysis.

While one of the limitations of the current study is that only MCI subjects have been assessed, we are aiming to recruit established AD patients to further validate the IRF parametric mapping strategy for ^11^C–PBR28 PET. Despite that, we have previously demonstrated that results gathered from a TSPO subgroup (HAB or MAB) can be translated to an entire AD and MCI population [16]. Another limitation is only HAB cases have been included; our group is recruiting more participants in order to evaluate the performance of ^11^C–PBR28 IRF in mixed binding affinity groups. SA is highly sensitive and is performed using arterial input analysis, which is the most robust quantitative method of analysis. Small variation could lead to bias in data estimation; however, the arterial blood acquisition and modelling in this study were carefully performed. Nevertheless, this preliminary report has revealed powerful evidence that parametric mapping of ^11^C–PBR28 using spectral analysis which separates the endothelial component provides a high-quality whole-brain map which could be used to analyze MCI subjects on an individual basis.

## Conclusion

In this study, we have demonstrated that spectral analysis can be used to generate ^11^C–PBR28 parametric maps. We have identified a high-frequency blood volume peak and three other spectral peaks which represent a slow irreversible trapping component and two tissue components. Results demonstrated ^11^C–PBR28 parametric maps generated by spectral analysis had a signal-to-noise ratio large enough to evaluate the microglial activation at voxel level on an individual basis. In conclusion, we have demonstrated, for the first time, that IRF parametric map generated by spectral analysis can reliably be used in quantifying ^11^C–PBR28 PET to assess neuroinflammation in prodromal Alzheimer’s disease.

## Electronic supplementary material

ESM 1 (DOCX 13 kb)

ESM 2 (DOCX 33 kb)

Online Resource 3 AlC values for 2TCM4k and 2TCM4k-1K compartmental model fitting (GIF 43 kb)

High resolution image (TIFF 603 kb)

Online Resource 4 Correlation between 11C-PBR28 IRF-90 and binding potential (BP_ND_) in frontal lobe, temporal lobe, parietal lobe and occipital lobe. (GIF 30 kb)

High resolution image (TIFF 372 kb)
