# Supplementary material for: Parametric mapping using spectral analysis for 11C-PBR28 PET reveals neuroinflammation in mild cognitive impairment subjects
Source: Eur J Nucl Med Mol Imaging. 2018 Mar 9;45(8):1432–41. doi: 10.1007/s00259-018-3984-5 (PMC5993844; doi:10.1007/s00259-018-3984-5)
Supplement: Supplementary file 1 — (DOCX 13 kb) [file 259_2018_3984_MOESM1_ESM.docx]

**Online Resource 1A. Coefficient of variation (CV) of IRF and Logan parametric maps**

|  | **CV of IRF** | | **CV of Logan** | |
| --- | --- | --- | --- | --- |
|  | MEAN | SD | MEAN | SD |
| Frontal Lobe | 20% | 5% | 21% | 3% |
| Temporal Lobe | 18% | 5% | 22% | 4% |
| Parietal Lobe | 18% | 4% | 20% | 3% |
| Occipital Lobe | 16% | 5% | 19% | 2% |
| Posterior Cingulate1 | 15% | 4% | 16% | 5% |
| Thalamus | 22% | 4% | 27% | 5% |
| Striatum | 26% | 4% | 33% | 5% |
| Hippocampus | 13% | 3% | 22% | 4% |
| Cerebellum | 20% | 4% | 22% | 4% |

**Online Resource 1B. Demographics**

| **Diagnosis group** | **TSPO binders** | **Age (SD)** | **Age range** | **MMSE (SD)** |
| --- | --- | --- | --- | --- |
| MCI | 13 | 70.9 (8.1) | 57 - 79 | 26.8 (2.9)* |
| HC | 9 | 65.4 (7.5) | 54 - 75 | 29.4 (1.0) |

*p<0.05
