# Supplementary material for: Parametric mapping using spectral analysis for 11C-PBR28 PET reveals neuroinflammation in mild cognitive impairment subjects
Source: Eur J Nucl Med Mol Imaging. 2018 Mar 9;45(8):1432–41. doi: 10.1007/s00259-018-3984-5 (PMC5993844; doi:10.1007/s00259-018-3984-5)
Supplement: Supplementary file 2 — (DOCX 33 kb) [file 259_2018_3984_MOESM2_ESM.docx]

**Online Resource 2A**. Single subject analysis. The corresponding significant clusters of ^11^C-PBR28 increase in MCI subjects compared to controls using IRF-90 parametric maps.

| **Region** | **Cluster Size** | | **MNI coordinates** | | | | |  | | **P(FWE-corr)** | | | **Z-score** | | |  |
| --- | --- | --- | --- | --- | --- | --- | --- | --- | --- | --- | --- | --- | --- | --- | --- | --- |
|  | (voxel) | | **x** | | **y** | | **z** | | | |  | | |  | | |
| **MCI 1** |  | |  | |  | |  | | | |  | | |  | | |
| Right Middle and inferior temporal gyrus | 110587 | | 35 | | -2 | | -28 | | | | 1.11E-16 | | | 4.08 | | |
| Right Lateral of occipital lobe |  | | 39 | | -79 | | -16 | | | |  | | | 4.07 | | |
| Right Superior frontal gyrus |  | | 15 | | -8 | | 56 | | | |  | | | 3.8 | | |
| Right insula |  | | 40 | | -2 | | -10 | | | |  | | | 3.78 | | |
| Right Lateral orbital gyrus |  | | 46 | | 29 | | -17 | | | |  | | | 3.77 | | |
| Right Superior parietal gyrus |  | | 6 | | -60 | | 17 | | | |  | | | 3.76 | | |
| Right Middlle frontal gyrus |  | | 39 | | 10 | | 53 | | | |  | | | 3.76 | | |
| Right Posterior temporal lobe |  | | 57 | | -34 | | -18 | | | |  | | | 3.74 | | |
| Left pallidum |  | | -18 | | 4 | | -4 | | | |  | | | 4.31 | | |
| Left Anterior temporal lobe, medial part | 35158 | | -33 | | 9 | | -38 | | | | 9.5E-07 | | | 4.03 | | |
| Left Posterior orbital gyrus |  | | -35 | | 30 | | -18 | | | |  | | | 3.81 | | |
| Left amygdala |  | | -27 | | -2 | | -22 | | | |  | | | 3.75 | | |
| Left Middlle frontal gyrus |  | | -31 | | 60 | | -6 | | | |  | | | 3.62 | | |
| Left Anterior temporal lobe, lateral part |  | | -42 | | 5 | | -36 | | | |  | | | 3.55 | | |
| Left Fusiform gyrus |  | | -37 | | -9 | | -40 | | | |  | | | 3.55 | | |
| Left hippocampus |  | | -21 | | -8 | | -25 | | | |  | | | 3.53 | | |
| Left Postcentral gyrus |  | | -39 | | -8 | | 15 | | | |  | | | 3.49 | | |
| **MCI 2** |  | |  | |  | |  | | | |  | | |  | | |
| Right Posterior temporal lobe | 6892 | | 49 | | -64 | | -12 | | | | 1.13E-06 | | | 4.89 | | |
| Right Lateral of occipital lobe |  | | 39 | | -79 | | -16 | | | |  | | | 4.63 | | |
| Left insula | 199551 | | -32 | | -7 | | 11 | | | | 0.0001 | | | 4.82 | | |
| Left Inferiolateral of parietal lobe |  | | -28 | | -70 | | 46 | | | |  | | | 4.72 | | |
| Left Lateral of occipital lobe |  | | -20 | | -72 | | 30 | | | |  | | | 4.7 | | |
| Left Postcentral gyrus |  | | -45 | | -18 | | 14 | | | |  | | | 4.5 | | |
| Left Lingual gyrus |  | | -14 | | -62 | | 3 | | | |  | | | 4.47 | | |
| Left thalamus |  | | -19 | | -33 | | 1 | | | |  | | | 4.65 | | |
| Left Posterior temporal lobe |  | | -50 | | -50 | | -7 | | | |  | | | 4.54 | | |
| Right Parahippocampal and ambient gyri |  | | 18 | | -15 | | -31 | | | |  | | | 4.7 | | |
| Right insula |  | | 32 | | -13 | | 7 | | | |  | | | 4.51 | | |
| Right thalamus |  | | 8 | | -20 | | 6 | | | |  | | | 4.41 | | |
| Right Inferiolateral of parietal lobe |  | | 64 | | -41 | | 39 | | | |  | | | 4.37 | | |
| Left Precentral gyrus | 7367 | | -43 | | 0 | | 28 | | | | 4.6E-07 | | | 4.17 | | |
| Left Middlle frontal gyrus |  | | -38 | | 9 | | 35 | | | |  | | | 4.02 | | |
| Left Inferior frontal gyrus |  | | -50 | | 31 | | 18 | | | |  | | | 3.72 | | |
| Right Superior frontal gyrus | 9538 | | 13 | | -11 | | 49 | | | | 9.25E-09 | | | 4.05 | | |
| Right Precentral gyrus |  | | 11 | | -32 | | 66 | | | |  | | | 3.86 | | |
| Right Postcentral gyrus |  | | 11 | | -30 | | 48 | | | |  | | | 3.72 | | |
| Right Gyrus cinguli, posterior part |  | | 11 | | -11 | | 44 | | | |  | | | 3.35 | | |
| Left Posterior orbital gyrus | 3397 | | -18 | | 9 | | -26 | | | | 0.0020 | | | 3.92 | | |
| Left Medial orbital gyrus |  | | -15 | | 19 | | -22 | | | |  | | | 3.89 | | |
| Left Straight gyrus |  | | -3 | | 20 | | -25 | | | |  | | | 3.67 | | |
| Left Subgenual frontal cortex |  | | -8 | | 25 | | -9 | | | |  | | | 3.46 | | |
| Right Medial orbital gyrus | 2760 | | 14 | | 34 | | -27 | | | | 0.0099 | | | 3.9 | | |
| Right Straight gyrus |  | | 11 | | 30 | | -18 | | | |  | | | 3.61 | | |
| Right Anterior orbital gyrus |  | | 15 | | 50 | | -17 | | | |  | | | 3.37 | | |
| Right Subgenual frontal cortex |  | | 7 | | 25 | | -8 | | | |  | | | 3.18 | | |
| Right Posterior orbital gyrus |  | | 25 | | 23 | | -19 | | | |  | | | 2.69 | | |
| **MCI 3** |  | |  | |  | |  | | | |  | | | 0 | | |
| Right Superior temporal gyrus, anterior part | 13974 | | 51 | | 21 | | -23 | | | | 0.0112 | | | 4.17 | | |
| Right Middle and inferior temporal gyrus |  | | 69 | | -12 | | -18 | | | |  | | | 4.14 | | |
| Right Inferior frontal gyrus |  | | 59 | | 25 | | 20 | | | |  | | | 4.1 | | |
| Right Postcentral gyrus |  | | 62 | | -15 | | 34 | | | |  | | | 3.66 | | |
| Right Middlle frontal gyrus |  | | 55 | | 18 | | 34 | | | |  | | | 3.21 | | |
| Right Inferiolateral of parietal lobe |  | | 65 | | -42 | | 39 | | | |  | | | 3.16 | | |
| Left Anterior temporal lobe, medial part | 12208 | | -34 | | 17 | | -31 | | | | 0.02824 | | | 3.52 | | |
| Left Superior temporal gyrus, posterior part |  | | -69 | | -22 | | 6 | | | |  | | | 3.21 | | |
| Left Inferiolateral of parietal lobe |  | | -65 | | -28 | | 41 | | | |  | | | 3.2 | | |
| **MCI 4** |  | |  | |  | |  | | | |  | | | 0 | | |
| Right cuneus | 208565 | | 7 | | -66 | | 15 | | | | 0.0001 | | | 4.48 | | |
| Right Lingual gyrus |  | | 13 | | -65 | | -6 | | | |  | | | 4.32 | | |
| Right Postcentral gyrus |  | | 62 | | -15 | | 35 | | | |  | | | 4.16 | | |
| Right Superior parietal gyrus |  | | 5 | | -60 | | 16 | | | |  | | | 4 | | |
| Right Inferiolateral of parietal lobe |  | | 64 | | -18 | | 32 | | | |  | | | 3.94 | | |
| Left Lingual gyrus |  | | -2 | | -75 | | -4 | | | |  | | | 4.38 | | |
| Left thalamus |  | | -17 | | -34 | | 0 | | | |  | | | 4.32 | | |
| Left Anterior temporal lobe, medial part |  | | -32 | | 9 | | -39 | | | |  | | | 4.07 | | |
| Left Precentral gyrus |  | | -31 | | -18 | | 72 | | | |  | | | 3.98 | | |
| Brainstem |  | | -6 | | -37 | | -17 | | | |  | | | 3.93 | | |
| **MCI 5** |  | |  | |  | |  | | | |  | | | 0 | | |
| Left insula | 51398 | | -32 | | -7 | | 11 | | | | 3.18E-09 | | | 4.62 | | |
| Left Posterior temporal lobe |  | | -50 | | -52 | | 10 | | | |  | | | 4.08 | | |
| Left Middle and inferior temporal gyrus |  | | -54 | | -29 | | -13 | | | |  | | | 3.9 | | |
| Left Lateral of occipital lobe |  | | -17 | | -87 | | 10 | | | |  | | | 3.65 | | |
| Left Superior parietal gyrus |  | | -4 | | -66 | | 21 | | | |  | | | 3.62 | | |
| Left putamen |  | | -22 | | 1 | | -1 | | | |  | | | 3.58 | | |
| Left Middlle frontal gyrus |  | | -18 | | 18 | | -10 | | | |  | | | 3.54 | | |
| Left Subgenual frontal cortex |  | | -4 | | 23 | | -12 | | | |  | | | 3.48 | | |
| Right Lingual gyrus |  | | 13 | | -65 | | -6 | | | |  | | | 3.85 | | |
| Right Middle and inferior temporal gyrus | 29148 | | 68 | | -12 | | -18 | | | | 1.12E-05 | | | 3.99 | | |
| Right Posterior temporal lobe |  | | 69 | | -44 | | -7 | | | |  | | | 3.95 | | |
| Right insula |  | | 40 | | -3 | | -11 | | | |  | | | 3.89 | | |
| Right Superior temporal gyrus, anterior part |  | | 50 | | 14 | | -20 | | | |  | | | 3.57 | | |
| Right Inferior frontal gyrus |  | | 54 | | 22 | | -5 | | | |  | | | 3.51 | | |
| Right Anterior temporal lobe, medial part |  | | 34 | | 12 | | -25 | | | |  | | | 3.31 | | |
| Right Inferiolateral of parietal lobe | 11122 | | 45 | | -41 | | 31 | | | | 0.0492 | | | 3.51 | | |
| Right Lateral of occipital lobe |  | | 47 | | -66 | | 23 | | | |  | | | 3.31 | | |
| Right Postcentral gyrus |  | | 24 | | -31 | | 43 | | | |  | | | 3.27 | | |
| Right Superior parietal gyrus |  | | 33 | | -36 | | 43 | | | |  | | | 2.88 | | |
| **MCI 6** |  | |  | |  | |  | | | |  | | | 0 | | |
| Left putamen | 354137 | | -19 | | 4 | | -5 | | | | 0.0001 | | | 4.75 | | |
| Left Posterior temporal lobe |  | | -51 | | -50 | | -7 | | | |  | | | 4.48 | | |
| Left hippocampus |  | | -21 | | -8 | | -24 | | | |  | | | 4.28 | | |
| Left Postcentral gyrus |  | | -3 | | -38 | | 58 | | | |  | | | 4.2 | | |
| Left Lingual gyrus |  | | -2 | | -76 | | -5 | | | |  | | | 4.15 | | |
| Left insula |  | | -34 | | 8 | | -6 | | | |  | | | 4.13 | | |
| Left Posterior orbital gyrus |  | | -29 | | 21 | | -23 | | | |  | | | 4.12 | | |
| Right cuneus |  | | 10 | | -74 | | 16 | | | |  | | | 4.33 | | |
| Right Inferior frontal gyrus |  | | 51 | | 42 | | -7 | | | |  | | | 4.28 | | |
| Right Inferiolateral of parietal lobe |  | | 52 | | -23 | | 22 | | | |  | | | 4.18 | | |
| Right thalamus |  | | 16 | | -29 | | -2 | | | |  | | | 4.17 | | |
| Right Middlle frontal gyrus |  | | 41 | | 56 | | 11 | | | |  | | | 4.12 | | |
| Right Medial orbital gyrus |  | | 13 | | 38 | | -20 | | | |  | | | 4.03 | | |
| Right Posterior temporal lobe |  | | 47 | | -61 | | 17 | | | |  | | | 4.02 | | |
| **MCI 7** |  |  | |  | |  | | |  | | |  | | |  |  |
| Right Posterior orbital gyrus | 363743 | 27 | | 28 | | -25 | | | 0.00001 | | | 5.27 | | |  |  |
| Right insula |  | 36 | | -9 | | -14 | | |  | | | 5.03 | | |  |  |
| Right Fusiform gyrus |  | 30 | | -10 | | -36 | | |  | | | 5.01 | | |  |  |
| Right Precentral gyrus |  | 20 | | -24 | | 52 | | |  | | | 4.7 | | |  |  |
| Right Superior parietal gyrus |  | 19 | | -54 | | 42 | | |  | | | 4.59 | | |  |  |
| Left insula |  | -33 | | -30 | | 4 | | |  | | | 5.26 | | |  |  |
| Left Posterior temporal lobe |  | -37 | | -39 | | -3 | | |  | | | 5.03 | | |  |  |
| Left Precentral gyrus |  | -34 | | -6 | | 40 | | |  | | | 4.92 | | |  |  |
| Left Middlle frontal gyrus |  | -23 | | 9 | | 40 | | |  | | | 4.9 | | |  |  |
| Left Postcentral gyrus |  | -33 | | -23 | | 33 | | |  | | | 4.89 | | |  |  |
| Corpus callosum |  | -3 | | 24 | | 11 | | |  | | | 4.6 | | |  |  |

**Online Resource 2B**. Single subject analysis. The corresponding significant clusters of [11C]PBR28 increase in MCI subjects compared to controls using Logan graphic VT parametric maps

| **Region** | **Cluster Size** | **MNI coordinates** | |  | **P(FWE-corr)** | **Z-score** |
| --- | --- | --- | --- | --- | --- | --- |
|  | (voxel) | **x** | **y** | **z** |  |  |
| **MCI 2** |  |  |  |  |  |  |
| Brainstem | 419157 | -4 | -18 | -9 | 0.00001 | 4.51 |
| Right Parahippocampal and ambient gyri |  | 28 | -21 | -30 |  | 4.35 |
| Right Middlle frontal gyrus |  | 32 | 40 | -4 |  | 4.2 |
| Right Postcentral gyrus |  | 50 | -8 | 12 |  | 4.12 |
| Right Superior frontal gyrus |  | 13 | 23 | 46 |  | 4.1 |
| Right Lateral orbital gyrus |  | 40 | 32 | -19 |  | 4.09 |
| Right Posterior temporal lobe |  | 65 | -36 | -19 |  | 4.09 |
| Right Anterior temporal lobe, medial part |  | 29 | 2 | -40 |  | 4.07 |
| Left Precentral gyrus |  | -12 | -14 | 52 |  | 4.22 |
| Left insula |  | -26 | 22 | -3 |  | 4.17 |
| Left Postcentral gyrus |  | -39 | -20 | 31 |  | 4.08 |
| Corpus callosum |  | 2 | 29 | 8 |  | 4.15 |
| **MCI 4** |  |  |  |  |  |  |
| Right Superior posterior temporal gyrus | 9282 | 38 | -8 | -20 | 0.0183 | 4.79 |
| Right insula |  | 43 | 8 | -9 |  | 3.56 |
| Right amygdala |  | 21 | -5 | -14 |  | 3.29 |
| Right Middlle frontal gyrus |  | 33 | 43 | -2 |  | 3 |
| Right Posterior orbital gyrus |  | 30 | 16 | -22 |  | 2.89 |
| Left amygdala |  | -19 | -6 | -16 |  | 2.88 |
| Brainstem | 11863 | -5 | -36 | -19 | 0.0026 | 4.43 |
| Left thalamus |  | -12 | -25 | 2 |  | 4.06 |
| Left hippocampus |  | -32 | -23 | -17 |  | 3.81 |
| Left Middle and inferior temporal gyrus |  | -45 | -11 | -20 |  | 3.3 |
| Left Superior anterior temporal gyrus | 86809 | -49 | 6 | -9 | 0.00001 | 4 |
| Left Lateral orbital gyrus |  | -49 | 43 | -12 |  | 3.87 |
| Left Posterior orbital gyrus |  | -35 | 31 | -22 |  | 3.65 |
| Left Precentral gyrus |  | -57 | -3 | 13 |  | 3.59 |
| Left Inferior frontal gyrus |  | -50 | 33 | -7 |  | 3.36 |
| Left Inferiolateral of parietal lobe |  | -53 | -30 | 48 |  | 3.18 |
| Right Precentral gyrus |  | 33 | -17 | 53 |  | 3.5 |
| Left Middlle frontal gyrus | 18575 | -22 | 35 | 5 | 0.00001 | 3.74 |
| Left Superior frontal gyrus |  | -15 | 14 | 29 |  | 3.18 |
| Right Pre-subgenual frontal cortex |  | 2 | 33 | -3 |  | 3.7 |
| Right Subgenual frontal cortex |  | 3 | 27 | -4 |  | 3.52 |
| Corpus callosum |  | -1 | 26 | 12 |  | 3.46 |
| **MCI 6** |  |  |  |  |  |  |
| Left insula | 246465 | -33 | 16 | 7 | 0.00001 | 3.95 |
| Left Inferior frontal gyrus |  | -38 | 3 | 23 |  | 3.92 |
| Left Straight gyrus |  | -1 | 21 | -17 |  | 3.8 |
| Left Middle and inferior temporal gyrus |  | -45 | -15 | -26 |  | 3.68 |
| Left Middlle frontal gyrus |  | -24 | 19 | 38 |  | 3.65 |
| Left Subgenual frontal cortex |  | -5 | 20 | -12 |  | 3.44 |
| Left Superior frontal gyrus |  | -19 | 47 | 22 |  | 3.42 |
| Left Precentral gyrus |  | -32 | -13 | 58 |  | 3.35 |
| Right Cingulate gyrus, anterior part |  | 10 | 35 | 16 |  | 3.95 |
| Right Superior frontal gyrus |  | 13 | 33 | -14 |  | 3.63 |
| Right Straight gyrus |  | 5 | 20 | -26 |  | 3.43 |
| Corpus callosum |  | 13 | 36 | 8 |  | 3.43 |
| Right Inferior frontal gyrus | 14975 | 60 | 14 | 7 | 0.0003 | 3.45 |
| Right insula |  | 32 | 6 | -14 |  | 3.28 |
| Right Postcentral gyrus |  | 49 | -7 | 6 |  | 3.06 |
| Right Middlle frontal gyrus |  | 47 | 54 | 6 |  | 2.65 |
| Right amygdala |  | 24 | -10 | -15 |  | 2.62 |
| Right putamen |  | 21 | 5 | -12 |  | 2.57 |
| **MCI 7** |  |  |  |  |  | 0 |
| Left Posterior temporal lobe | 14909 | -51 | -49 | -7 | 0.0066 | 3.64 |
| Left Inferiolateral of parietal lobe |  | -39 | -61 | 29 |  | 3.34 |
| Left Middle and inferior temporal gyrus |  | -56 | -29 | -15 |  | 3.3 |
| Left Superior temporal gyrus |  | -53 | -28 | 3 |  | 2.83 |
| Left hippocampus |  | -31 | -28 | -11 |  | 2.81 |
| Right Middlle frontal gyrus | 11260 | 20 | 42 | 10 | 0.045 | 3.64 |
| Right Precentral gyrus |  | 33 | -7 | 33 |  | 3.41 |
| Right insula |  | 33 | -10 | 7 |  | 3.1 |
| Right Anterior orbital gyrus |  | 28 | 43 | -11 |  | 2.88 |
| Right Postcentral gyrus |  | 34 | -17 | 20 |  | 2.83 |
