# Supplementary figures and images for: Parametric mapping using spectral analysis for 11C-PBR28 PET reveals neuroinflammation in mild cognitive impairment subjects
Source: Eur J Nucl Med Mol Imaging. 2018 Mar 9;45(8):1432–41. doi: 10.1007/s00259-018-3984-5 (PMC5993844; doi:10.1007/s00259-018-3984-5)

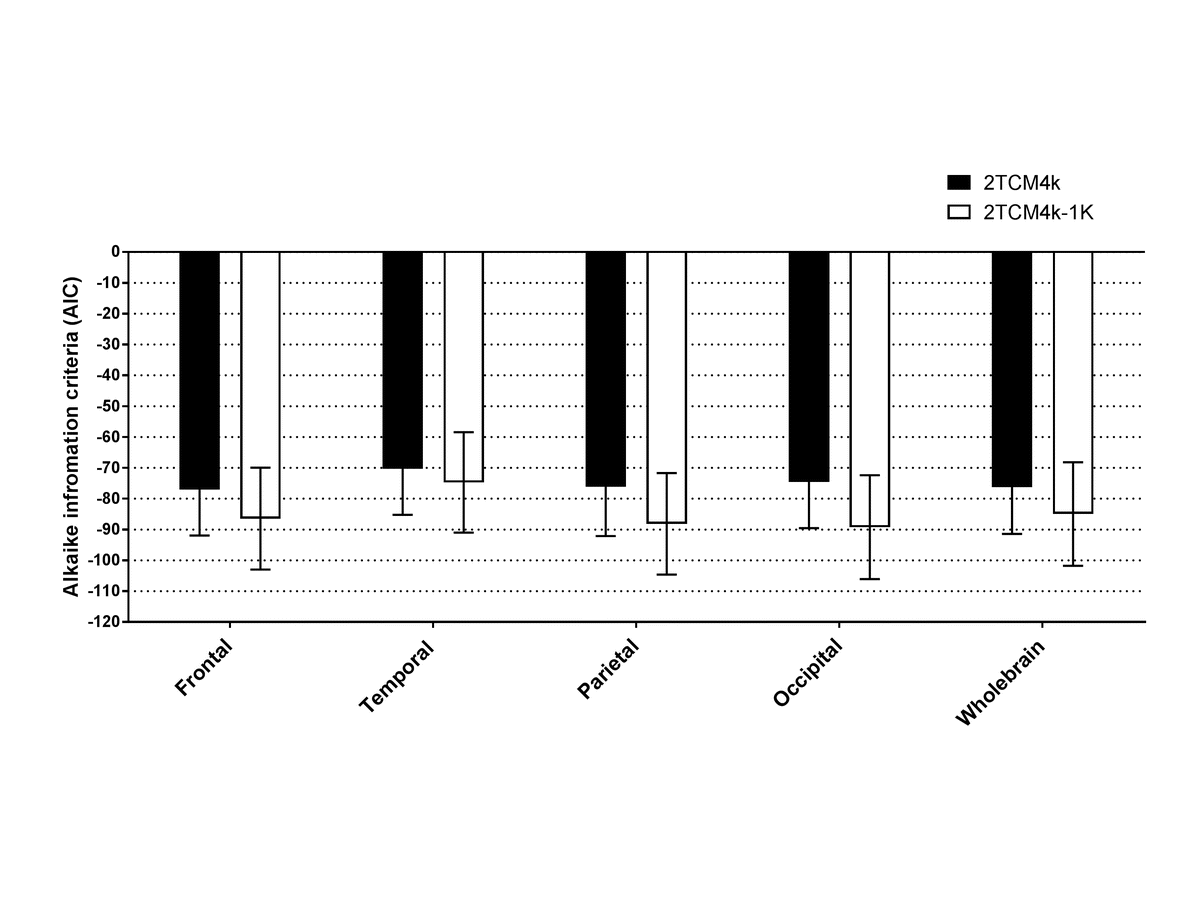

Supplement: Supplementary file 3 — AlC values for 2TCM4k and 2TCM4k-1K compartmental model fitting (GIF 43 kb) [file 259_2018_3984_Fig6_ESM.gif]

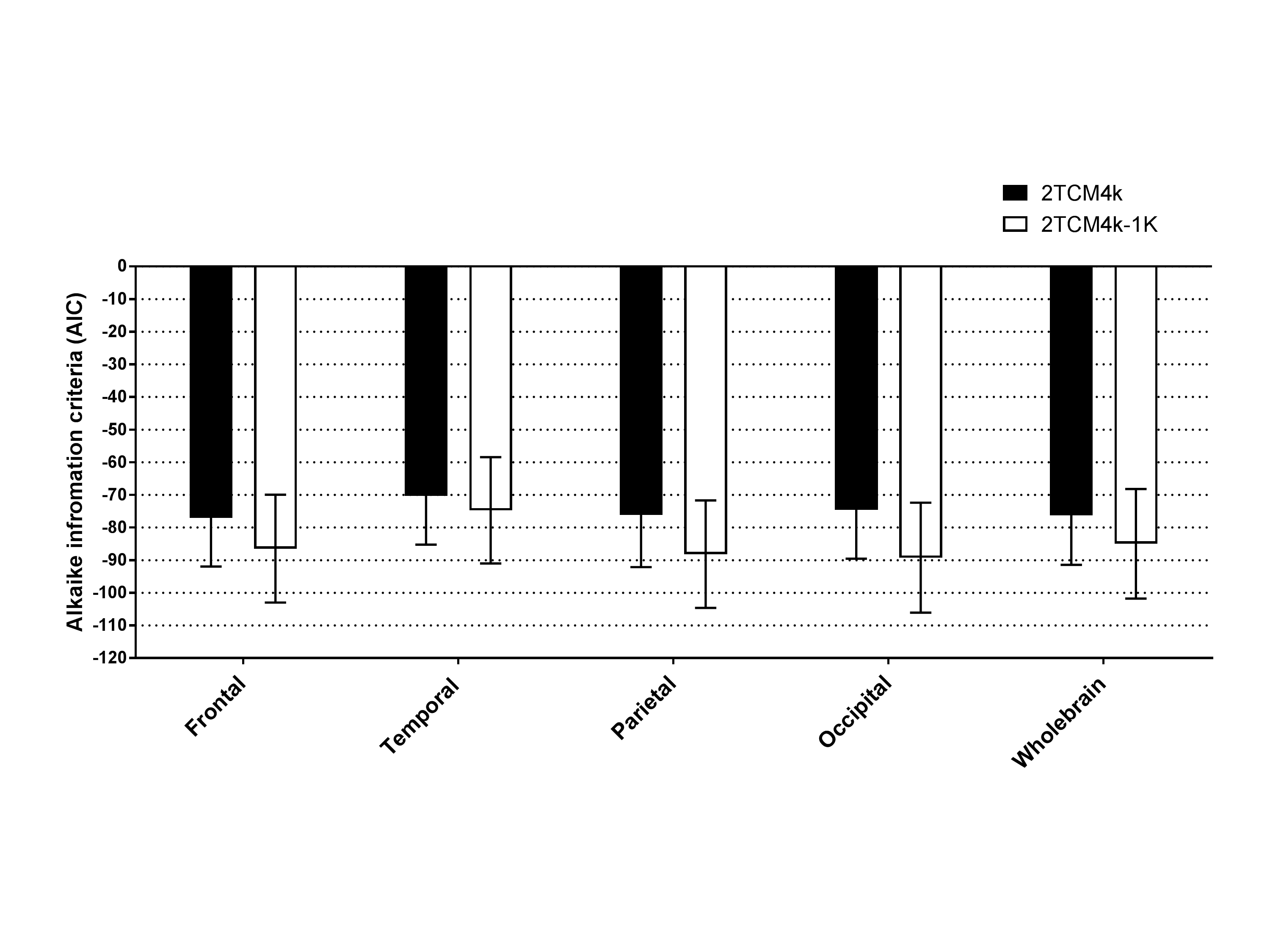

Supplement: Supplementary file 4 — High resolution image (TIFF 603 kb) [file 259_2018_3984_MOESM3_ESM.tif]

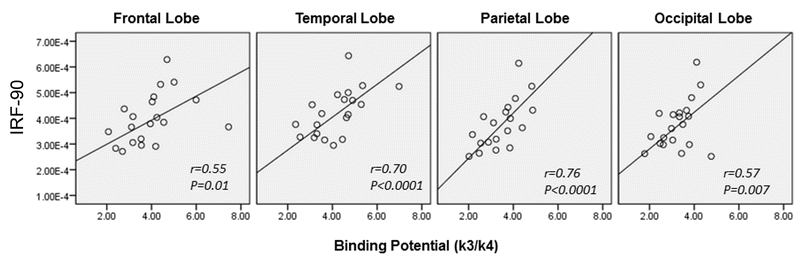

Supplement: Supplementary file 5 — Correlation between 11C-PBR28 IRF-90 and binding potential (BPND) in frontal lobe, temporal lobe, parietal lobe and occipital lobe. (GIF 30 kb) [file 259_2018_3984_Fig7_ESM.gif]

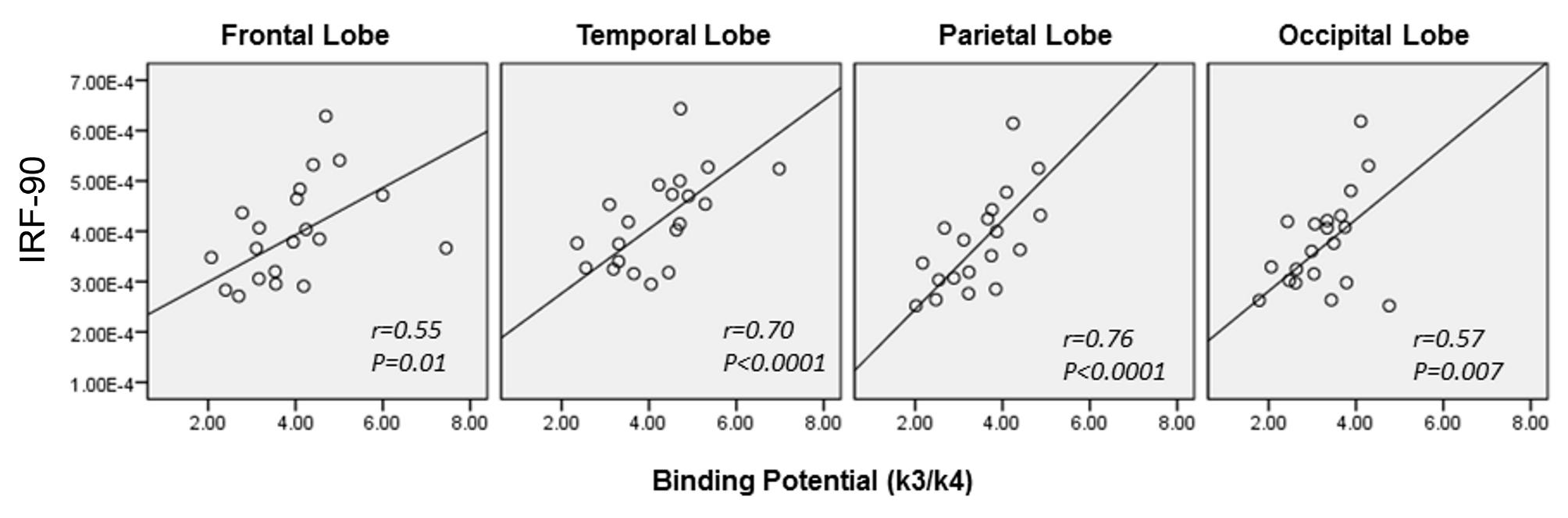

Supplement: Supplementary file 6 — High resolution image (TIFF 372 kb) [file 259_2018_3984_MOESM4_ESM.tif]
